# Identifying appropriate protected areas for endangered fern species under climate change

**DOI:** 10.1186/s40064-016-2588-4

**Published:** 2016-06-27

**Authors:** Chun-Jing Wang, Ji-Zhong Wan, Zhi-Xiang Zhang, Gang-Min Zhang

**Affiliations:** School of Nature Conservation, Beijing Forestry University, Beijing, 100083 China

**Keywords:** Conservation area, Endangered fern species, Climate change, Species distribution modeling, Conservation planning software, China

## Abstract

The management of protected areas (PAs) is widely used in the conservation of endangered plant species under climate change. However, studies that have identified appropriate PAs for endangered fern species are rare. To address this gap, we must develop a workflow to plan appropriate PAs for endangered fern species that will be further impacted by climate change. Here, we used endangered fern species in China as a case study, and we applied conservation planning software coupled with endangered fern species distribution data and distribution modeling to plan conservation areas with high priority protection needs under climate change. We identified appropriate PAs for endangered fern species under climate change based on the IUCN protected area categories (from Ia to VI) and planned additional PAs for endangered fern species. The high priority regions for protecting the endangered fern species were distributed throughout southern China. With decreasing temperature seasonality, the priority ranking of all endangered fern species is projected to increase in existing PAs. Accordingly, we need to establish conservation areas with low climate vulnerability in existing PAs and expand the conservation areas for endangered fern species in the high priority conservation regions.

**Electronic supplementary material:**

The online version of this article (doi:10.1186/s40064-016-2588-4) contains supplementary material, which is available to authorized users.

## Background

Climate change has had a profound effect on biodiversity and can result in the migration, adaption, and extinction of species, as well as make it harder to protect endangered species (Pearson and Dawson 2003; Hampe and Petit 2005; Dawson et al. 2011; Chen 2013). Some studies have shown that changes in population and species structure may alter distributions of species diversity, affect habitats and thus induce responses in the phenotypic plasticity of individuals and populations, and change the distribution or fragmentation of habitats (Jackson and Sax 2010; Sgro et al. 2011; Zhang et al. 2014a; Chung et al. 2015). This ultimately reduces species diversity and results in a loss of biodiversity (Sgro et al. 2011; Bradford and Warren 2014).

Ferns are vascular plants that reproduce and disperse via spores (Graf 1999). A number of endangered fern species (EFS) have been seriously impacted by climate change and are in danger of extinction. However, current protection measures do not adequately support the conservation of EFS (http://www.iucnredlist.org/). Therefore, EFS conservation efforts are urgently required under climate change.

These protection issues can be addressed by the establishment of additional protected areas (PAs; Chape et al. 2005; Chen 2007). The future effectiveness of PAs is limited because climate change could drive endangered plant species out of PAs, resulting in a loss of their conservation function for endangered species (Araújo et al. 2011; Yu et al. 2014; Wang et al. 2016). A number of studies have suggested the integration of climate change into conservation planning for endangered plant species (Hannah et al. 2002; Araújo et al. 2011; Dawson et al. 2011). However, studies focusing on EFS conservation in conjunction with climate change are rare. Target seven of the Global Strategy for Plant Conservation (GSPC) has shown at least 75 % of known threatened plant species would be conserved in situ from 2011 to 2020 (https://www.cbd.int/gspc/). However, conservationists and government managers cannot effectively establish more PAs for EFS because of limited funds and manpower (Leader-Williams and Albon 1988; Zhang et al. 2014a). Hence, it is necessary for conservationists to expand PAs to accommodate EFS, assess the ability of existing protected areas to conserve EFS, and determine the climatic features of these PAs. Thus, we aimed to determine the areas and number of wild EFS populations through field investigations and to plan to appropriately expand protected areas for EFS.

Conservation planning software and species distribution modeling (SDM) has been widely used in biological conservation, ecological restoration, and the planning of PAs (Summers et al. 2012; Chen 2013; Meller et al. 2014). Researchers have predicted the potential geographical distributions of endangered species, determined priority conservation areas for these species, and established a model-based evaluation system for the conservation of biodiversity in PAs by using conservation planning software and SDM (Di Minin and Moilanen 2012; Summers et al. 2012; Di Minin and Moilanen 2014; Wan et al. 2016). Conservation planning software coupled with SDM could be used to identify appropriate protected areas for EFS and determine priority conservation areas under climate change that are not covered by existing PAs (Di Minin et al. 2013).

We used the EFS of China as a case study because (1) EFS are widely distributed across a range of latitudes, (2) China contains a rich diversity of EFS, and (3) EFS conservation management is urgent because there are few PAs supporting EFS in China. The main objective of our study was to identify appropriate conservation areas for EFS under climate change based on conservation priority rankings computed with conservation planning software. To achieve this objective, we performed two tasks: (1) an evaluation of the ability of PAs to conserve EFS under climate change using Zonation (a common conservation planning software tool) and (2) a determination of the climatic features of PAs with high priority rankings. First, we used SDM in Maxent to model the potential distribution of EFS in China under climate change. Second, we used Zonation to plan priority conservation areas for EFS based on this potential distribution. Third, geographical information system (GIS) was used to compute the ability of protected areas to conserve EFS under climate change and explore the relationship between climate change and priority conservation areas in PAs.

## Methods

### PAs in China

Data from the World Database on Protected Areas (WDPA) were used to identify the PAs in China with areas greater than 4.3 km at the equator that were suitable for analysis in this study (http://www.protectedplanet.net/). We classified 642 Chinese PAs into five groups based on the IUCN protected area categories: Category Ia, strict nature reserve; Category II, national park; Category IV, habitat/species management area; Category V, protected landscape/seascape; and Category VI, protected area with sustainable use of natural resources. Based on WDPA database, there are no PAs belonging to Category Ib (wilderness area) and Category III (natural monument or feature; http://www.protectedplanet.net/).

### Species data

EFS were selected from the List of National Key Protected Wild Plants approved by the State Council of China (http://www.gov.cn/gongbao/content/2000/content_60072.htm). Occurrence localities that contain EFS were identified from the following three sources: (1) The Global Biodiversity Information Facility (GBIF; http://www.gbif.org/); (2) the Chinese Virtual Herbarium (CVH; http://www.cvh.org.cn/); and (3) 175 scientific research reports detailing national nature reserves (more detailed information is provided in the “Acknowledgements” section). Although some studies did not report the geographical coordinates of some species, we were able to translate the recorded locations of species into latitudes and longitudes using Google Earth and ArcGIS 10.2 (Esri; Redlands, CA, USA) based on (1) the detailed location and habitat descriptions of species from the research reports; (2) vegetation information about species from the 1:1 Million Vegetation Atlas of China (Hou 2001); and (3) the locations of species within 10-arc-minute grid cells (equivalent to 16 km at the equator) to avoid any georeferencing errors (Zhang et al. 2014b). These three factors limited the extent of species occurrences to roughly 10-arc-minute grids. Finally, we selected 16 EFS with more than five occurrence localities as the input dataset for SDM (Pearson et al. 2007). We could not identify wild populations of some species owing to limited occurrence localities. For practical purposes, we focused on potential distributions of EFS with known wild populations.

### Modeling potential distributions of species

Four contemporary bioclimatic variables at a 10-arc-minute spatial resolution (16 km at the equator) were used to model potential distributions of species, and these climatic data were obtained from the WorldClim database (Additional file 1: Table S1; http://www.worldclim.org/). The resulting four bioclimatic variables were related to the distribution and physiological performance of plants. Four projected bioclimatic variables, corresponding to the present-day variables, were assessed using the mean grid maps of three global climate models (GCMs), including mohc_hadgem2, csiro_mk3_6_0, and cccma_canesm2 analogue data (corresponding to 2070–2099, or roughly the 2080 s), and obtained from the International Centre for Tropical Agriculture (http://ccafs-climate.org). Representative concentration pathways (RCPs) 4.5 (mean, 780 ppm; range, 595–1005 by 2100; low concentration scenario) and 8.5 (mean, 1685 ppm; range, 1415–1910 by 2100; high concentration scenario) were used to model future potential distributions of species. RCP 8.5 projections differ from RCP 4.5 projections because of higher cumulative concentrations of carbon dioxide and other largely anthropogenic greenhouse gas pollutants that alter the pattern of climate change (http://www.ipcc.ch/report/ar5/).

We used Maxent to model the projected EFS distributions in China under climate change (Merow et al. 2013). All grids were regarded as a possible distribution space according to maximum entropy (Elith et al. 2011; Merow et al. 2013). For the map grids predicted using Maxent, cell values of one represented the highest possibility of containing the species, while values close to zero represented the lowest possibility. Furthermore, projected EFS distribution areas were effectively determined using the contemporary climate conditions of the present-day sites that contain these individual EFS (Warren and Seifert 2011).

Maxent settings used in this analysis included the following: (1) the regularization multiplier (beta) was set to 1.5 for producing a smooth and general response that could be modeled in a biologically realistic manner (Shcheglovitova and Anderson 2013); (2) the maximum number of background points was 10,000 (Merow et al. 2013); (3) a fourfold cross-validation approach was used for removing bias with respect to recorded occurrence points, namely, 75 % of occurrence points were used for training and 25 % for the actual test (Li and Guo 2013); (4) a jackknife test was used in Maxent to analyze the importance of different climatic factors (Merow et al. 2013); and (5) all other settings used were the same as those described by Elith et al. (2011) and Merow et al. (2013). We projected the importance of climatic variables to potential distributions of species based on the results of the jackknife test (Merow et al. 2013).

Receiver operating characteristic (ROC) curves summarize each value of the prediction result as a possible analysis threshold. The precision of the model was evaluated by calculating the area under the ROC Curve (AUC). The models were either graded as poor (AUC < 0.8), fair (0.8 < AUC < 0.9), good (0.9 < AUC < 0.95), or very good (0.95 < AUC < 1.0; Adhikari et al. 2012). Using the methods described by Calabrese et al. (2014), we computed predicted species richness under current, low, and high concentration scenarios by superimposing the weighted potential distribution of species for each grid. Then we selected some PAs that currently contain EFS (based on scientific surveys conducted in national nature reserves) and used linear-regression analyses to analyze the relationship between mean predicted species richness of grids and observed species richness in PAs in order to evaluate model precision at the PA scale. A significant relationship between these values is an important precondition for computing the priority ranking of the EFS. Here, we could not use all of the PAs from WDPA because of a lack of data. Hence, we only included the PAs for which species occurrence data was recorded in all data sources (i.e., GBIF, CVH, and scientific research reports by national nature reserves).

### Evaluating the ability of PAs to conserve EFS

First, we used Zonation software to identify priority conservation areas for EFS in China. Zonation is a publicly available framework and software for grid-based and large-scale spatial conservation prioritization (Meller et al. 2014). It has been used for evaluating conservation areas and conservation planning under climate change scenarios (Summers et al. 2012). Using Zonation, we obtained the priority ranking of each grid for EFS. We focused on the connectivity between the current and future potential EFS distributions (under the low and high concentration scenarios) and considered the influence of climate change on future species richness when selecting potential sites for nature reserves (Lehtomäki and Moilanen 2013; Wan et al. 2015). Using the original core-area cell removal rule, we established spatial priorities and calculated the marginal loss of each grid, which we then used to determine if a conservation goal had been met (i.e., that a given proportion of distributions for all of the species with the high priority ranking would be protected; Lehtomäki and Moilanen 2013; Wang et al. 2015). Current and future species richness of EFS were weighted equally in our analysis (including under low and high concentration scenarios), and we used a warp factor of 100 (Wan et al. 2014, 2015; Wang et al. 2015). For the grid maps of priority conservation areas under low and high concentration scenarios, 10.0-arc-minutes resolution data were aggregated at 2.5-arc-minutes resolution using ArcGIS 10.2 (Esri; Redlands, CA, USA) to identify priority conservation areas in PAs. We also used ArcGIS 10.2 (Esri; Redlands, CA, USA) to extract and compute the priority ranking of all EFS and the average values of the most important climatic variables for PAs.

We computed the overall priority ranking of all EFS for each PA using the following equation:

$$S_{t} = {{\sum\limits_{j = 1}^{n} {P_{j} } } \mathord{\left/ {\vphantom {{\sum\limits_{j = 1}^{n} {P_{j} } } A}} \right. \kern-0pt} A}$$

where *S*_*t*_ represents the overall priority ranking of all EFS in PA *t*, *P*_*j*_ represents the priority ranking value of all EFS in grid *j* based on PA *t*, and *A* represents the number of grids in PA *t*.

Finally, we used linear-regression analyses to compute the relationship between the average values of the most important climatic variables and priority ranking of all EFS for PAs. We also computed the mean priority ranking of all EFS for each group of PAs based on the IUCN protected area.

## Results

**Fig. 1:**
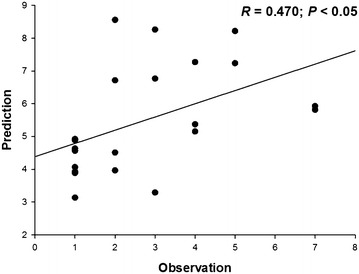
The relationship between mean predicted species richness of grids and observed species richness at the scale of protected areas. *Prediction* mean predicted species richness of grids; *Observation* observed species richness

**Fig. 2:**
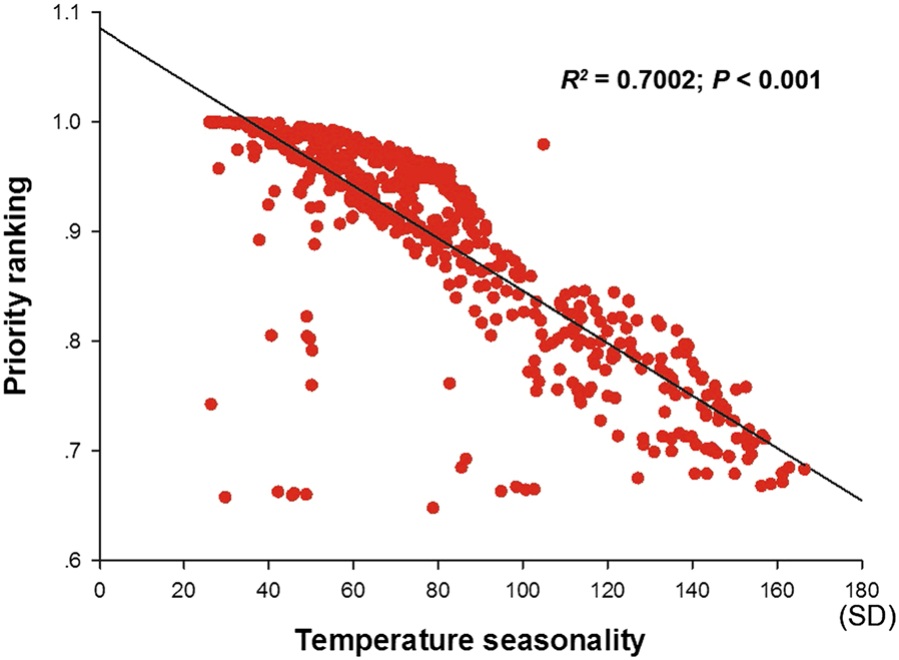
The relationship between priority rankings of endangered fern species and temperature seasonality. *Priority ranking* the priority ranking of a single endangered fern species

We predicted the potential distributions of 16 EFS in China under current, low, and high concentration scenarios (Additional file 2: Fig. S1). Based on AUC values, our prediction model performed well for all species: the AUC value of each species was over 0.9 (mean value, 0.974; Table 1). We also found that there was a significant relationship between the mean predicted species richness of grids and the observed species richness in PAs (*R* = 0.470; *P* < 0.05; Fig. 1). Based on the jackknife test in Maxent, we found that the most important climatic variable was temperature seasonality (average contribution to EFS distributions, 59.8; Table 1). Furthermore, there was a significant relationship between priority ranking of all EFS and temperature seasonality (*R* ^2^ = 0.7002; *P* < 0.001; Fig. 2) indicating that with decreasing temperature seasonality the priority ranking of all the EFS would increase in PAs (Fig. 2).

**Table 1:** Endangered fern species, AUC values, and jackknife test results

Names	Family	AUC	Bio1	Bio4	Bio12	Bio15
*Cibotium barometz*	Dicksoniaceae	0.978	4.0	49.8	25.3	20.8
*Archangiopteris henryi*	Angiopteridaceae	0.996	99.1	0.9	0.0	0.0
*Sorolepidium glaciale*	Dryopteridaceae	0.976	0.6	58.3	0.0	41.2
*Helminthostachys zeylanica*	Helminthostachyaceae	0.989	1.0	73.6	0.1	25.3
*Isoetes sinensis*	Isoetaceae	0.965	0.2	2.5	9.8	87.5
*Ceratopteris thalictroides*	Parkeriaceae	0.947	16.7	46.3	24.2	12.8
*Neocheiropteris palmatopedata*	Polypodiaceae	0.992	0.0	93.4	6.6	0.0
*Alsophila costularis*	Cyatheaceae	0.992	0.0	92.5	0.1	7.4
*Alsophila denticulata*	Cyatheaceae	0.967	2.3	43	29	25.7
*Alsophila gigantea*	Cyatheaceae	0.984	1.6	63.2	7.2	28
*Alsophila loheri*	Cyatheaceae	0.999	0.0	94.7	5.3	0.0
*Alsophila metteniana*	Cyatheaceae	0.967	3.0	51.9	29.9	15.2
*Alsophila podophylla*	Cyatheaceae	0.959	1.5	65.9	26.2	6.3
*Alsophila spinulosa*	Cyatheaceae	0.967	1.4	66.8	28.3	3.5
*Sphaeropteris lepifera*	Cyatheaceae	0.964	0.4	88.8	10.4	0.4
*Brainea insignis*	Blechnaceae	0.942	1.9	65.9	25.8	6.5
Mean		0.974	8.4	59.8	14.3	17.5

**Fig. 3:**
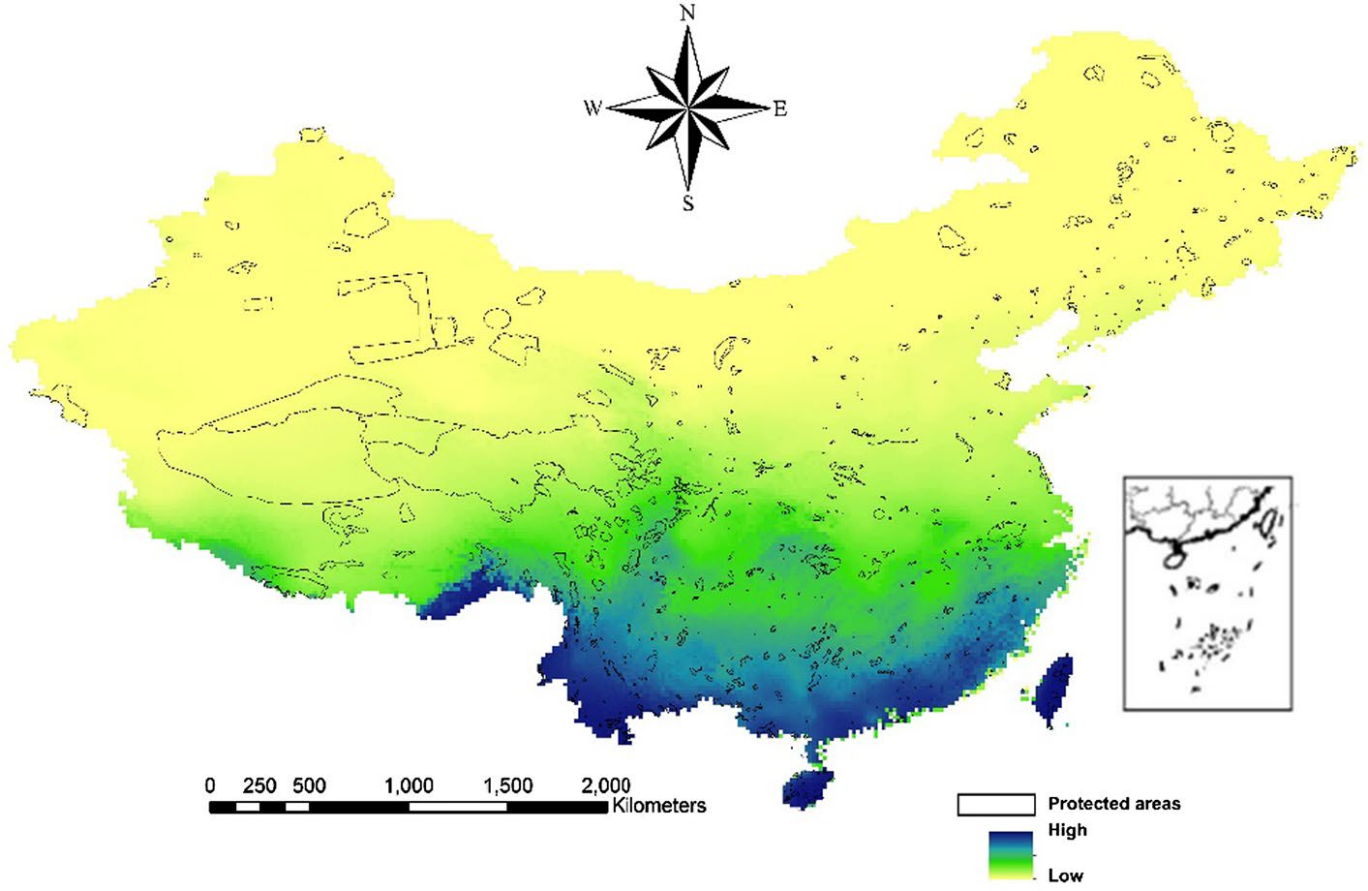
A priority ranking map for endangered fern species in China. The *color* distribution from *light* to *dark* represents increasing priority ranking

**Fig. 4:**
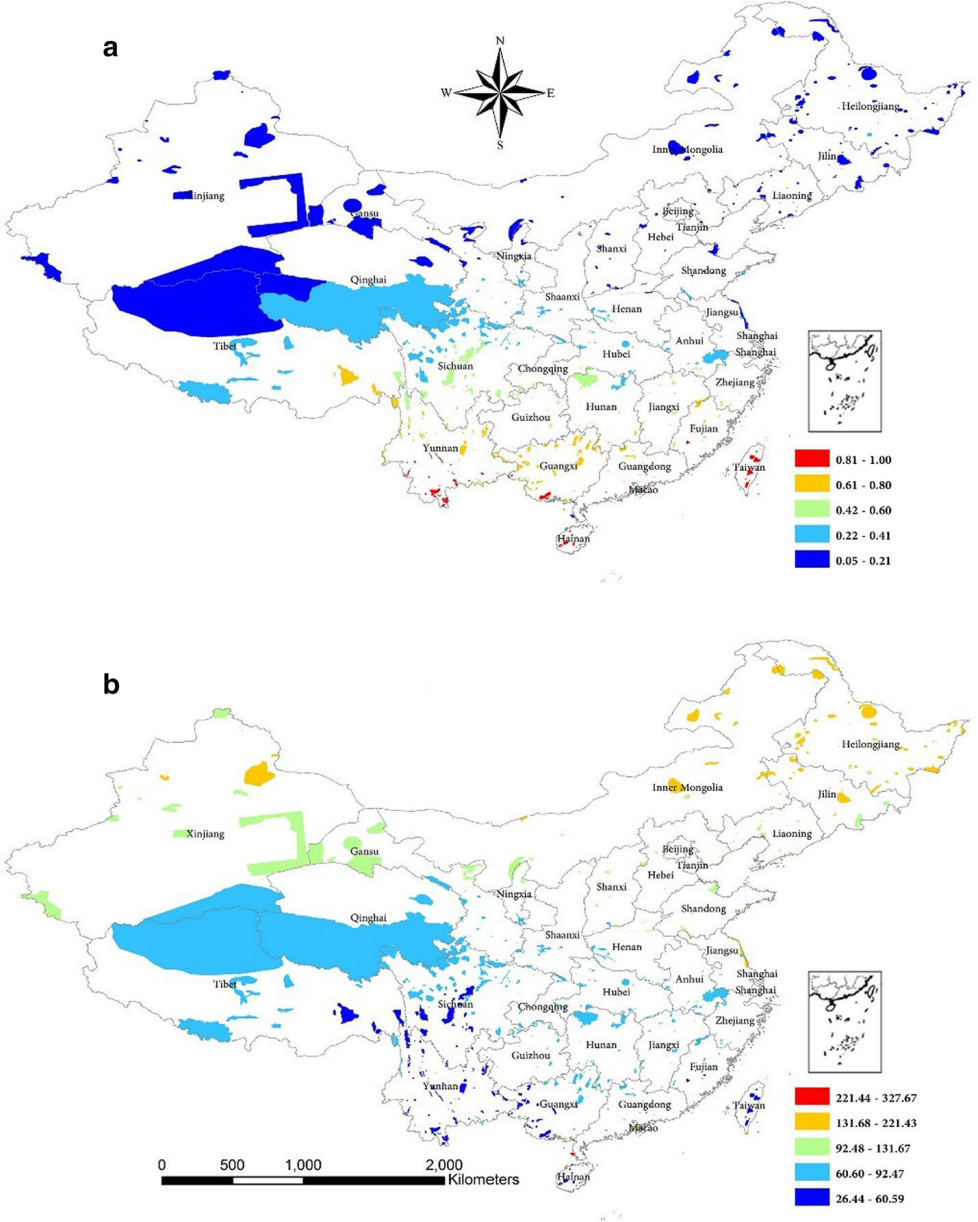
Maps showing **a** priority ranking and **b** temperature seasonality of protected areas. The *color* distribution from *blue* to *red* represents increasing **a** priority ranking and **b** temperature seasonality

**Fig. 5:**
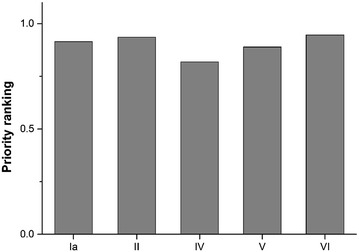
The priority ranking of protected areas based on IUCN protected area categories. *Priority ranking* the mean priority ranking of endangered fern species in China

We found that the regions with high priority ranking for all EFS were distributed throughout southern China (Fig. 3). This is consistent with the present-day distribution of occurrence localities of species we collected. Tawushan (Sichuan), Taitung Hungyeh Village Taiwan Cycas (Taiwan), Yushan (Taiwan), Chuyunshan (Taiwan), and Nanlin (Hainan) PAs had high priority rankings for all EFS (Fig. 4a). These PAs are projected to effectively converse EFS. Furthermore, these PAs exhibit low temperature seasonality (Fig. 4b). We found that national parks and protected areas that permit the sustainable use of natural resources have a higher priority ranking for all EFS than strict nature reserves, habitat/species management areas, and protected landscapes/seascapes (Fig. 5). The PAs of habitat/species management areas have the lowest priority rankings for all EFS (Fig. 5).

## Discussion

This study establishes a large-scale evaluation system for EFS based on the predicted impact of climate change. We suggest the use of robust results of potential species distributions to identify appropriate protected areas for EFS under different climate change models. To achieve this objective, we used two test methods for the potential EFS distributions. First, AUC provides important references that were used to assess the performance of Maxent, and then we tested the robustness of predicted species richness modeled by Maxent at the PA scale based on the relationship between predicted species richness and observed species richness of PAs (Pouteau et al. 2015). Previous studies have shown that the relationship between the predicted species richness of grids and the observed species richness is robust at the grid scale (Royle et al. 2012; Cao et al. 2013; Pouteau et al. 2015). However, the limits of occurrence locality data could not perfectly support this test method at the grid scale (Chen 2013). Hence, in addition to our main objectives, we also validated that the predicted species richness based on projected species distributions was robust at the PA scale.

Conservationists and government managers have begun to integrate climate change into conservation management (Lawler 2009; Heller and Zavaleta 2009; Dawson et al. 2011). This is likely a consequence of the increased understanding that climate change could drive potential distributions of plant species out of existing PAs, such that these PAs could lose their function of conserving endangered species (Araújo et al. 2011). Previous studies have shown that the assessment of the ability of existing PAs to conserve endangered plant species can be an effective reference for enhancing the conservation of endangered plant species (Araújo et al. 2011; Wan et al. 2014; Yu et al. 2014; Wang et al. 2016). Accordingly, action is required to establish conservation areas of low climate vulnerability for EFS in existing PAs (Gillson et al. 2013). However, the conservation functions of many existing Chinese PAs are primarily intended to protect forest ecosystems and endangered animals (http://datacenter.mep.gov.cn/). There is a substantial opportunity to utilize these existing PAs to conserve EFS. We should therefore focus on national parks and protected areas that exhibit a sustainable use of natural resources in China. National parks are similar to wilderness areas in size and in their ecosystem protection function. Protected areas that sustainably use natural resources are focused on establishing mutually beneficial arrangements for nature conservation and the sustainable management of natural resources (http://www.iucn.org/). However, these two types of PAs are affected by human disturbance. Hence, in situ conservation areas should be separated from areas with a high density of human activities (Ravenel and Redford 2005; Wang et al. 2015). The PA priority rankings indicate we have selected appropriate PAs—i.e., Tawushan (Sichuan), Taitung Hungyeh Village Taiwan Cycas (Taiwan), Yushan (Taiwan), Chuyunshan (Taiwan) and Nanlin (Hainan)—for conducting detailed EFS investigations and enhancing in situ EFS conservation. In order to continue supporting EFS, these PAs need to have low temperature seasonality. Hence, it is necessary to integrate projected changes in temperature seasonality into the future EFS conservation efforts.

Some studies have suggested the expansion of PAs to allow for distribution changes under climate change (Heller and Zavaleta 2009; Lawler 2009; Araújo et al. 2011; Dawson et al. 2011). However, limited manpower and financial resources constrain the development of additional PAs (Leader-Williams and Albon 1988; Wang et al. 2016). Hence, we need to identify appropriate protected areas for EFS under climate change and develop more protected areas for EFS efficiently (Chen 2007; Heller and Zavaleta 2009). Our results shown in Figs. 3 and 4 indicate the need to establish a network of PAs that facilitate the exchange of EFS among PAs under climate change; conservation planning software could address this need (Di Minin and Moilanen 2012; Summers et al. 2012; Di Minin and Moilanen 2014). Sharafi et al. (2012) used Zonation to identify areas outside of existing PAs that efficiently cover gaps in biodiversity features and appropriately expand conservation areas in Victoria, Australia. This study provides a useful model for conservation efforts in China. Our suggestion is to strongly consider connectivity among existing PAs and to establish conservation areas for EFS in the regions among PAs with high priority rankings.

## Conclusion

Our findings show that with decreasing temperature seasonality, national parks and protected areas in which natural resources are sustainably used had the highest priority ranking for all of the analyzed EFS. To reduce the negative impact of climate change on EFS, we should take immediate actions, such as establishing conservation areas with low climate vulnerability for EFS in existing PAs and expanding conservation areas for EFS into the regions with high priority rankings. We hope to use a large-scale priority ranking evaluation for the areas that are suitable for EFS conservation in order to promote the development of global conservation planning for threatened plant species. However, our study had some limitations, as we required more detailed data on climate and species distributions than was sometimes available. In future research, we will construct more accurate maps of appropriate conservation areas for EFS under climate change with appropriately richer data. Immediate EFS conservation actions should be considered in future worldwide studies.

## Additional files

**Additional file 1: Table S1.** Environmental variables. Environmental variables were used as environmental layers to characterize the current distribution and predict the potential distribution of endangered fern species using Maxent; C of V represents the coefficient of variation; SD represents Standard Deviation.

**Additional file 1: Fig. S1.** The contemporary and projected distributions of endangered fern species in the (a) current, (b) low, and (c) high concentration scenarios.
