# Supplementary material for: Identifying appropriate protected areas for endangered fern species under climate change
Source: Springerplus. 2016 Jun 27;5(1):904. doi: 10.1186/s40064-016-2588-4 (PMC5434847; doi:10.1186/s40064-016-2588-4)
Supplement: Supplementary file 1 — Additional file 1: Table S1. Environmental variables. Environmental variables were used as environmental layers to characterize the current distribution and predict the potential distribution of endangered fern species using Maxent; C of V represents the coefficient of variation; SD represents Standard Deviation. [file 40064_2016_2588_MOESM1_ESM.docx]

**Table S1. Environmental variables.** Environmental variables were used as environmental layers to characterize the current distribution and predict the potential distribution of endangered fern species using Maxent; C of V represents the coefficient of variation; SD represents Standard Deviation.

| **Code** | **Environmental variables** | **Unit** |
| --- | --- | --- |
| Bio1 | Annual mean temperature | °C |
| Bio4 | Temperature seasonality | SD |
| Bio12 | Annual precipitation | mm |
| Bio15 | Precipitation seasonality | C of V |
