# Supplementary material for: Identifying appropriate protected areas for endangered fern species under climate change
Source: Springerplus. 2016 Jun 27;5(1):904. doi: 10.1186/s40064-016-2588-4 (PMC5434847; doi:10.1186/s40064-016-2588-4)
Supplement: Supplementary file 2 — Additional file 1: Fig. S1. The contemporary and projected distributions of endangered fern species in the (a) current, (b) low, and (c) high concentration scenarios. [file 40064_2016_2588_MOESM2_ESM.docx]

**Fig. S1. The contemporary and projected distributions of endangered fern species in the (a) current, (b) low, and (c) high concentration scenarios.**

**
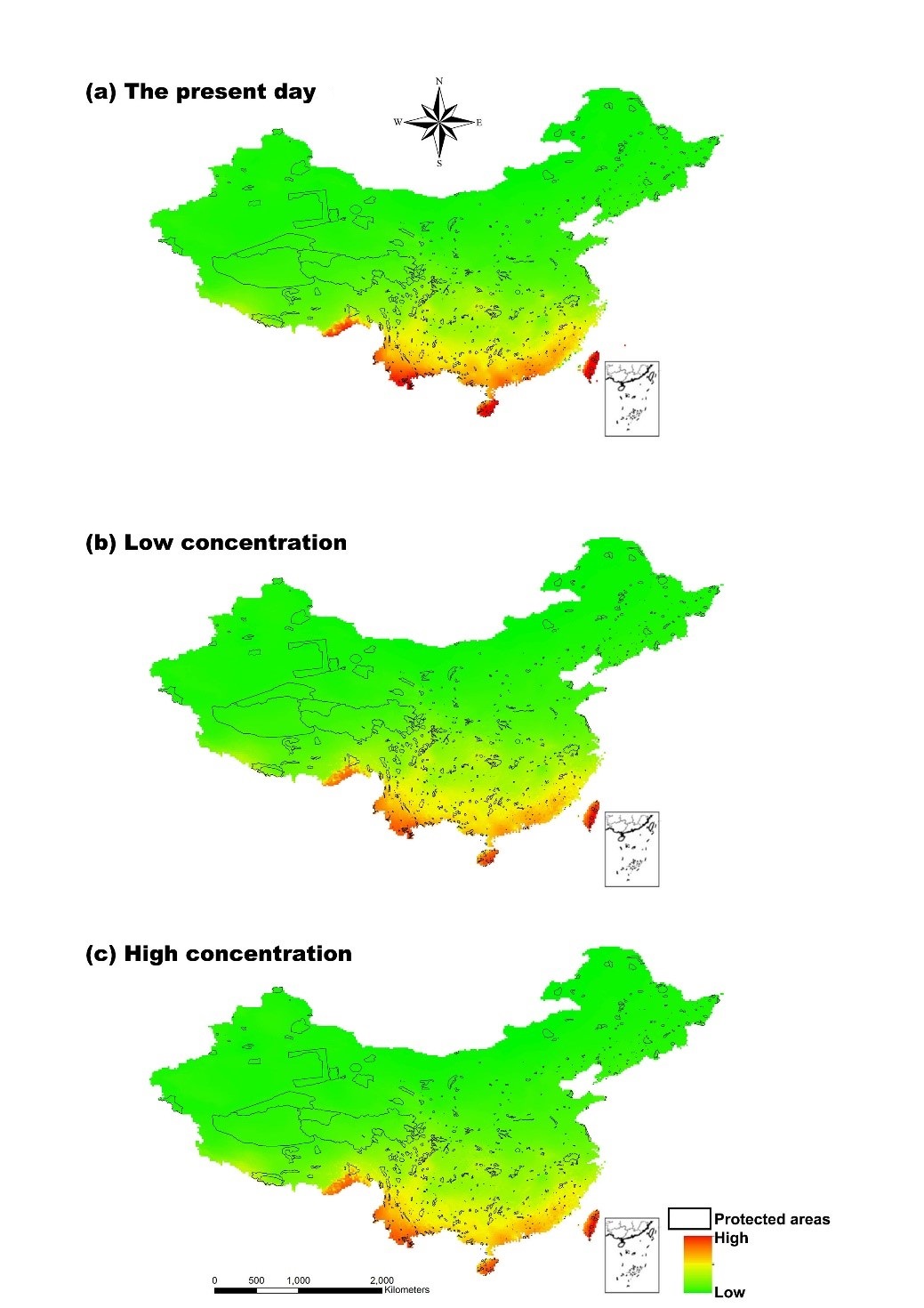
**
